# Retraction: A 12,800-year-old layer with cometary dust, microspherules, and platinum anomaly recorded in multiple cores from Baffin Bay

**DOI:** 10.1371/journal.pone.0342613

**Published:** 2026-02-11

**Authors:** The PLOS One Editors

Following publication of this article [1], concerns were raised regarding referencing, methodology, and data reporting in this article. The *PLOS One* Editors have consulted multiple experts regarding these concerns. The following issues with this article have been identified:

- Incorrect citations following an issue with the DOI information provided by the authors, raising concerns about undisclosed use of AI tools.
- Multiple citations are incorrectly used and do not support the associated statements made.
- Several concerns have been raised about the reported chronology. These relate to (a) issues with reporting of the methods used to generate new dates; and (b) issues with calibration leading to potentially erroneously older ages. Concerns raised include, but are not limited to:
  - Missing information regarding the foraminifera species or type used for dating, and the number of individuals used.
  - Lack of clarity on how authors accounted for the uncertainty of producing dates from 5 cm sediment layers.
  - The method for calibrating the radiocarbon ages does not follow the recommended methods for the latitude of the study site and time period. Calibrating radiocarbon ages using the recommended methods produces ages that are younger than those reported in the article and that are outside the Younger Dryas.
  - It is unclear if presented ages are calibrated or modelled.
- Misidentification of material in SI Figs S30–S34 (https://zenodo.org/records/14681287). The authors claim these are spherule fragments identified as cosmic impact indicators. During expert consultation, these have been identified as marine foraminifera. The presence of foraminifera will affect the Ca curves presented and raise concerns with the peak abundance of cosmic material identified in each core, as some of this may be from foraminifera presence. S32 and S34 have clearly visible micropores and a chemical composition of Ca, C, O. Furthermore, it is unknown how much of the reported ‘Cosmic indicator spike’ includes this misidentified material.
- Sampling methodology does not allow for long term variability in cosmic particle concentration to be assessed. The peak at core 64 of a single data point is outside the presented Younger Dryas Boundary, but of the same magnitude as the peak at the Younger Dryas Boundary in core 67.
- The article does not position itself within wider literature, with no discussion as to why the mainstream hypothesis is refuted.
- The data in the Zenodo data repository is not always clearly attributed.

In light of the above concerns that call into question the validity of the reported results and the conclusions made, the *PLOS One* Editors retract this article. PLOS regrets that the issues were not identified prior to the article’s publication.

JJ agreed with the retraction. CM, JK, AA, KL, SC, GK, AW, and ML did not agree with the retraction. VAT responded but expressed neither agreement nor disagreement with the editorial decision. DM, MB, WN, MB, VA, SS, JP, TW, MY, MA, RG, and JM either did not respond directly or could not be reached.

## References

[1] Moore CR, Tselmovich VA, LeCompte MA, West A, Culver SJ, Mallinson DJ, et al. RETRACTED: A 12,800-year-old layer with cometary dust, microspherules, and platinum anomaly recorded in multiple cores from Baffin Bay. PLoS One. 2025;20(8):e0328347. doi: 10.1371/journal.pone.0328347 40768403 PMC12327676

